# Rituximab-associated hypogammaglobulinemia in children with idiopathic nephrotic syndrome: results of an ESPN survey

**DOI:** 10.1007/s00467-023-05913-1

**Published:** 2023-04-04

**Authors:** Aleksandra Zurowska, Magdalena Drozynska-Duklas, Rezan Topaloglu, Antonia Bouts, Olivia Boyer, Mohan Shenoy, Marina Vivarelli, on behalf of ESPN Glomerulonephritis Working Group, H. Alpay, R. Andersen, G. Ariceta, B. Atmış, U. S. Bayrakçı, B. Esrea, V. Baudouin, N. Bervina, E. Benetti, E. Berard, A. Bjerre, M. Christian, A. Couderc, J. Dehoorne, G. Deschenes, C. Dossier, R. Düşünsel, Z. Ekinci, F. Emma, L. Espinoza, A. Gianviti, M. Herrero Goñi, G. Guido, L. Ghio, J. Groothoff, D. Ö. Hacıhamdioğlu, M. Espino Hernández, A. Jankauskiene, M. Kagan, M. Kemper, M. Kovacevic, S. Kohl, R. T. Kramar, M. López-González, A. M. Aris, S. Maringhini, M. Marlais, M. Melgosa, A. Mitsioni, G. Montini, A. Moczulska, L. Murer, V. Obukhova, J. Oh, P. Ortega, Z. B. Özçakar, T. Ulinski, A. Pasini, T. Papalia, S. Paunova, A. Pena, C. Pecoraro, E. Petrosyan, L. Peruzzi, N. Printza, L. Prikhodina, C. Pietrement, S. Rittig, D. Rodrigo, N. Savenkova, M. Saraga, F. L. Sever, M. Schreuder, M. Szczepanska, V. Tasic, B. Tonshoff, K. Tullus, J. Vara, J. Vande Walle, E. Volokhina, J. Zieg, A. Waters, L. T. Weber, N. Webbe, M. Wasiak

**Affiliations:** 1 Department of Pediatrics, Nephrology and Hypertension, Medical University of Gdańsk, ul. Debinki 7, 80-952 Gdańsk, Poland; 2 Centre for Rare Diseases, Medical University of Gdańsk, Gdańsk, Poland; 3 Department of Pediatric Nephrology, Hacettepe University School of Medicine Hacettepe University, Ankara, Turkey; 4 Department of Pediatric Nephrology, Emma Children’s Hospital, Amsterdam University Medical Centers, Amsterdam, The Netherlands; 5 Department of Pediatric Nephrology, Reference Center for Idiopathic Nephrotic Syndrome in Children and Adults, Necker Hospital, Paris, France; 6 Laboratory of Hereditary Kidney Diseases, Imagine Institute, Paris Descartes University, Paris, France; 7 Department of Paediatric Nephrology, Royal Manchester Children’s Hospital, Manchester University Hospitals NHS Foundation Trust, Manchester Academic Health Science Centre, Manchester, UK; 8 Division of Nephrology and Dialysis, Bambino Gesù Children’s Hospital and Research Institute, Rome, Italy

**Keywords:** Rituximab, Hypogammaglobulinemia, Nephrotic syndrome, Children

## Abstract

**Background:**

There is paucity of information on rituximab-associated hypogammaglobulinemia (HGG) and its potential infectious consequences in children treated for idiopathic nephrotic syndrome (INS).

**Methods:**

A survey was distributed by the European Society Pediatric Nephrology to its members. It addressed the screening and management practices of pediatric nephrology units for recognizing and treating RTX-associated HGG and its morbidity and mortality. Eighty-four centers which had treated an overall 1328 INS children with RTX responded.

**Results:**

The majority of centers administered several courses of RTX and continued concomitant immunosuppressive therapy. Sixty-five percent of centers routinely screened children for HGG prior to RTX infusion, 59% during, and 52% following RTX treatment. Forty-seven percent had observed HGG prior to RTX administration, 61% during and 47% >9 months following treatment in 121, 210, and 128 subjects respectively. Thirty-three severe infections were reported among the cohort of 1328 RTX-treated subjects, of whom 3 children died. HGG had been recognized in 30/33 (80%) of them.

**Conclusions:**

HGG in steroid-dependent/frequently relapsing nephrotic syndrome (SDNS/FRNS) children is probably multifactorial and can be observed prior to RTX administration in children with SDNS/FRNS. Persistent HGG lasting >9 months from RTX infusion is not uncommon and may increase the risk of severe infections in this cohort. We advocate for the obligatory screening for HGG in children with SDNS/FRNS prior to, during, and following RTX treatment. Further research is necessary to identify risk factors for developing both HGG and severe infections before recommendations are made for its optimal management.

**Graphical abstract:**

**Figure:**
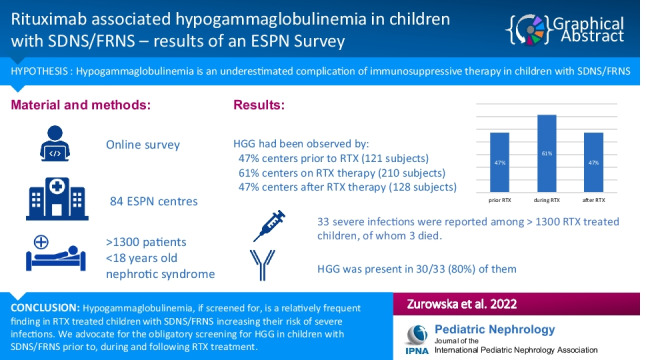
A higher resolution version of the Graphical abstract is available as Supplementary information

**Supplementary Information:**

The online version contains supplementary material available at 10.1007/s00467-023-05913-1.

## Introduction

Rituximab (RTX) is a monoclonal antibody against CD20, an antigen expressed on the surface of all circulating B cells excluding plasma cells. Licensed for the treatment of malignancies, vasculitis, and rheumatoid arthritis, it is used off label for the treatment of different autoimmune disease and has been used increasingly for the management of idiopathic nephrotic syndrome (INS) mainly in children with poorly controlled steroid-dependent/frequently relapsing nephrotic syndrome (SDNS/FRNS) [1–3]. Recent randomized controlled trials have confirmed its efficacy for this indication, but paucity of data exist on its optimal dosage and short- and long-term safety [4–10]. Targeting B cells should be the rationale for using RTX in INS, nevertheless evidence exists that RTX can also impact T cells [11, 12]. Depletion of CD20-expressing B cells in the peripheral blood following RTX infusion is rapid and long lasting (several months). Though anti-CD20 antibodies were not expected to influence immunoglobulin levels, the association of RTX use and hypogammaglobulinemia (HGG) has been well established in adults, less so in the pediatric population [13, 14]. A number of risk factors including RTX dose, concomitant immunosuppressive (IMS) treatment, and pre-existing low immunoglobulin levels seem to play a role in its development [15–19]. The reported incidence in adults is relatively low, ranging from 14–20% in adults with malignancies to 3.5–4.2% in subjects with autoimmune diseases [18, 20–22]. The clinical significance of this acquired HGG is not fully known but recent publications have shown an increased risk of infections following its use in adults and children [18, 19, 23]. Due to the paucity of data on the incidence of HGG and its consequences in RTX-treated children with INS, a survey was undertaken by the Glomerulonephritis Working Group (WG) of the European Society Pediatric Nephrology (ESPN) addressing the screening and management practices in place in pediatric nephrology centers for the recognition and treatment of this complication and its potential association with severe infections.

## Materials and methods

An online survey was distributed by the Glomerulonephritis WG to all ESPN members by email. The survey was web based and developed in the English language. It assessed three domains: center policy for administration of RTX (dose, number of courses, co-administrated drugs, CD19/20 monitoring), screening policy for HGG defined as serum levels below age-standardized reference ranges (prior, during and after 9 months after RTX infusion), its management with IgG replacement therapy (IGRT), and RTX-associated morbidity and mortality (center observations on the number of patients with severe infections requiring hospitalization, data on individual etiology and outcome). The survey consisted of 15 main questions and 25 subquestions of which 29 were close-ended and 11 open-ended. Respondents were able to review and change their answers. Question response types included 20 dichotomous, 8 single choice, 1 multiple choice, and 11 free text responses. The survey was developed by means of Google Documents Application. Participants were informed on the number of questions and time for survey completion. The survey was non-obligatory and no incentives were offered. Answers were stored automatically in a database.

Replies were obtained from 84 centers representing 14 EU countries (Belgium, Czech, Denmark, France, Germany, Greece, Italy, Malta, the Netherlands, Poland, Portugal, Spain, Sweden, and the UK), 2 non-EU countries (Russia and Turkey), and 3 non-European locations (Canada, Israel, and Iran). The response rate was 100% for 28 queries and between 91 and 98% for the remaining 13. Data analysis was performed using STATISTICA (Stat Soft. Inc). The survey results are presented in accordance with the CHERRIES reporting guidelines [24].

## Results

Eighty-forty centers reported treating a total number of 1328 children with RTX for steroid-sensitive INS. RTX was given to children with SDNS/FRNS who were poorly controlled with standard steroid and IMS therapy.

### Center policies for rituximab administration

The majority (22/84) of centers prescribed RTX independently of age, 23 limited RTX use to children > 5 years and 8 to subjects > 10 years age. RTX was given as a single infusion (375 mg/m^2^) by 51/84 (60%) centers and as two consecutive infusions by 28/84. Twenty-three units prescribed RTX as a single course of treatment, 44 gave 1–5 courses, and 17 centers had given > 5 RTX courses. The majority (46/61; 75%) routinely assessed CD19/20 counts during treatment, adapting further RTX infusions according to CD19/CD20 reconstitution. Steroids were continued by 47/84 (56%) centers and IMS drugs by 59/84 (70%). Only 15 units discontinued all concomitant treatment with the introduction of anti-CD20 therapy (Table 1).

**Table 1:** Center policies for RTX treatment of idiopathic nephrotic syndrome

Number of centers	84 (100%)
Initial dosing of RTX	
Single dose 375 mg/m^2^ Double dose 375 mg/m^2^ Other	51 [60%] 28 [31%] 7 [8%]
Number of maintenance RTX courses	
Single course Multiple courses Dosing dependent on CD19/CD20	23 [27%] 61 [72%] 46/61 [75%]
CD19/20 monitoring	
Adjustment of further RTX courses by CD19/20 levels Fixed timing of further RTX courses	46/61 [75%] 15/61 [25%]
Age restriction for RTX administration	
Administration independent of age Restricted to children >2 years og age Restricted to children >3 years of age Restricted to children >5 years of age Restricted to children >10 years of age	22/84 [26%] 23/84 [27%] 8/84 [9%] 23/84 [27%] 8/84 [9%]
Concomitant IMS therapy	
Steroids CNI MMF None	47/84 [56%] 41/84 [49%] 48/84 [57%] 15/84 [18%]

### Recognition of hypogammaglobulinemia

**Fig. 1:**
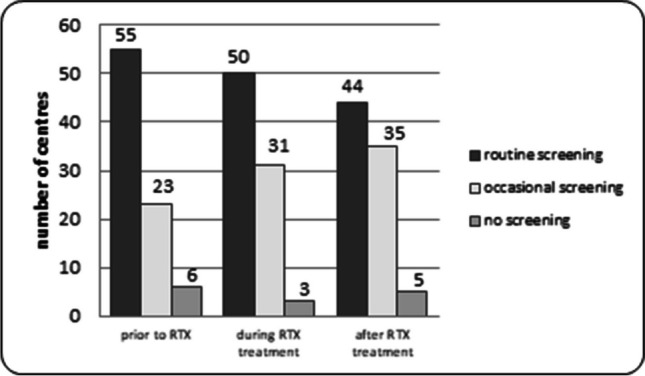
Screening policies for hypogammaglobulinemia in SDNS/FRNS children treated with rituximab in 84 European centers (pre, during, and post treatment)

Prior to initial RTX infusion 78/84 centers checked serum IgG levels, 55 routinely (65%). Following RTX administration 81/84 centers checked serum IgG, 50 routinely (59%). Late screening of IgG levels was performed by 79/84 centers, 44 routinely (52%) (Fig. 1).

**Fig. 2:**
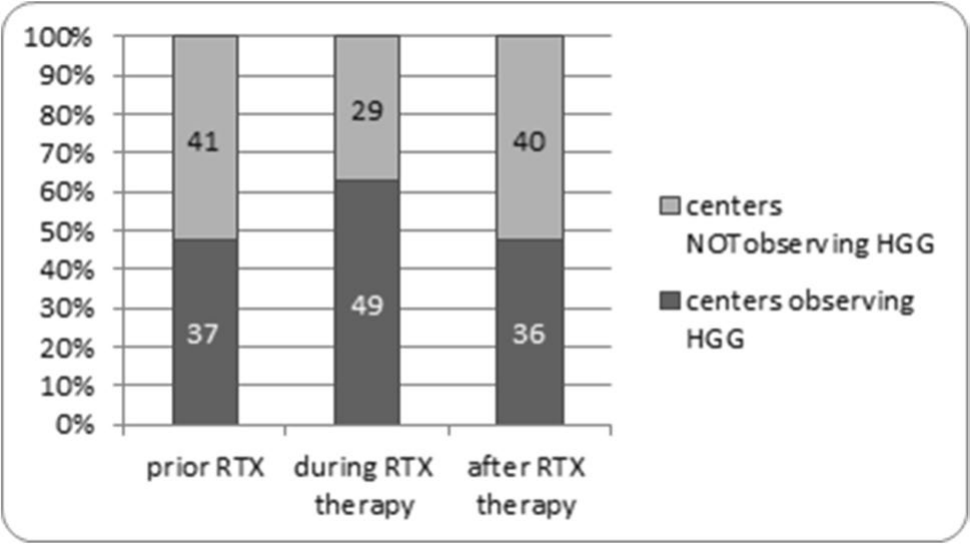
Number of centers reporting hypogammaglobulinemia in RTX-treated children with SDNS/FRNS. Survey results from 84 European pediatric nephrology centers

Nearly half (47%) of the actively screening units (37/78) reported observing HGG in patients prior to RTX administration in a cumulative number of 121 children. Sixty-one percent of actively screening units (49/78) observed HGG in children during RTX treatment, reporting a total number of 210 children with this complication. Forty-seven percent of units (36/76) declared they had observed persistent HGG (> 9 months after RTX infusion) in a total number of 128 children (Fig. 2).

Anti-CD20 antibodies were administered by 28/37 (75%) centers despite preexisting HGG, 19 of them routinely prescribing prophylactic IGRT. During RTX treatment, 30/49 centers (62%) prescribed prophylactic IgG to some of their patients, 19 never used them. When persistent HGG was recognized 10/35 centers routinely administered prophylactic IgG, the majority 17/35 (49%) supplementing IgG to individual patients and 8 centers left HGG untreated. In spite of persistent HGG, 81% (29/36) centers continued IMS or steroid therapy.

### Morbidity and mortality associated with RTX in children with INS

**Fig. 3:**
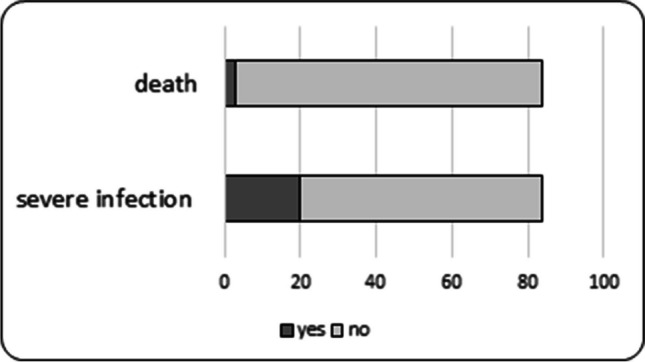
Number of centers reporting severe infections (morbidity) and death from severe infections (mortality) in children with SDNS/FRNS treated with RTX. Severe infections reported by 20/84 centers in 33/over 1300 children treated with RTX; deaths reported by 3/82 centers in 3/ over 1300 children treated with RTX

Twenty out of eighty-four centers (23%) observed severe infections in children associated with the use of RTX (Fig. 3). Thirty-three severe infections requiring hospitalization were reported among the overall 1328 RTX-treated subjects. The most frequently noted were upper respiratory tract infections and pneumonias of pneumococcal, pneumocystis, and viral etiology (12), followed by sepsis (4) and a variety of viral (myocarditis, herpes infections, meningoencephalitis, measles) and bacterial infections (skin, mastoiditis, pertussis, neuroborreliosis, urinary tract). The majority of children (30/33) demonstrated low serum IgG levels (80%). Three deaths were reported, two due to respiratory tract infections (streptococcus pneumonia, RSV pneumonia) and one due to pneumonia and sepsis of unknown etiology (Table 2).

**Table 2:** Clinical data of 33 severe infections reported among 1328 rituximab-treated children with idiopathic nephrotic syndrome

Severe infection requiring hospitalization	Number reported	Etiology	Number reported	mortality
Pneumonia	12	Str.pneumoniae Pneumocystis RSV unknown	4 3 1 4	1 0 1 0
Sepsis	4	Str.pneumoniae Unknown	2 2	0 1
Other probable bacterial infections:		Unknown		0
Skin infections/cellulitis Mastoiditis Staphylococcal infection Pertussis *Neuroborreliosis* Intestinal Urinary tract infection	3 1 1 1 1 2 1	Unknown Unknown Staphylococcus Bordetellapertussis Borrelia Unknown unknown		0 0 0 0 0 0 0
Other viral infections:				
Myocarditis Herpes infections (one eye infection) Meningoencephalitis Measles	2 3 1 1	Unknown Herpes zoster Enterovirus Measles virus		0 0 0 0

## Discussion

The ESPN survey on the presence of HGG in RTX-treated children with INS collected information from 84 pediatric nephrology centers treating an overall number of 1328 children, the largest cohort of children subjected to this off-label therapy reported to date. The presented results include the majority of European centers using anti-CD20 antibodies for the treatment of childhood INS, as access to this type of therapy is not universal [25]. The presented results are therefore representative of the contemporary attitudes of mainly European pediatric nephrologists for screening and recognizing HGG in RTX-treated nephrotic subjects.

The reported center policies for RTX administration were surprisingly uniform in spite of lacking consensus guidelines and limited published clinical trials on its use in children for this indication. Only 9% of centers used it solely for older children, the remaining administrating it without age restriction or in children >5 years of age. Concern in prescribing anti-CD20 antibodies to very young children is justified due to the lack of data on its direct and possible long-term effect on their immunological system. Younger age at RTX administration has been reported to be associated with the occurrence of HGG and poorer response to treatment [26, 27]. The protocol most frequently used by centers was cautious with 60% administering a single dose of 375 mg/m^2^. The majority of units administered more than one course of treatment, reflecting the practice of offering anti-CD20 therapy to children with poorly controlled SDNS/FRNS who demonstrate relapses following previous IMS therapy and also relapsed following RTX use [28]. The frequently observed (82% centers) use of concomitant IMS therapy and/or steroids with RTX increased the children’s risk of developing acquired immunodeficiency.

Routine assessment of immunoglobulins prior to RTX administration was frequent but not a universal practice (2/3 of centers). The center-based design of our survey did not enable an exact calculation of the incidence of HGG in RTX-treated children with SDNS/FRNS. Nevertheless, 47% of pediatric nephrology units had observed pre-RTX low IgG levels in a total number of 121 patients. Data from a large cohort of adults treated for malignancies or autoimmune diseases suggests it may be relatively common as it was noted in nearly half of the screened 655 subjects [18]. The causes of pretreatment HGG have been related to previous IMS/steroid treatment, a previously undiagnosed primary common variable immunodeficiency or urinary loss of immunoglobulins [15, 16, 18]. Both primary and secondary causes of HGG have been recognized as risk factors for the severity of RTX-associated HGG and its related infectious complications [16, 17, 19, 22, 23, 29]. The increased rate of infectious complications in RTX-treated adults with rheumatoid arthritis led to the publication of a consensus statement for the pretreatment assessment of Ig in this disease population [30, 31]. Similarly, we advocate for the routine assessment of immunoglobulin levels prior to initiation of anti-CD20 therapy in INS children. Although 50% of centers reported a policy of prophylactic IGRT, there is currently no consensus regarding its use. The decision may be personalized depending on the age of the patient, vaccine competency, previous history of infections, and possibly severity of HGG.

Transient HGG following RTX infusion is a recognized phenomenon though its incidence is not well described [14, 32]. In most patients, RTX does not reduce immunoglobulin levels significantly as it does not target the antigen specific IgG-producing plasma cells. Between 24 and 34% of adults treated with RTX develop HGG [33, 34]. The survey results and recently published data from a Japanese study suggest that it may have a similar or even higher incidence in children [35]. The management of this complication was not uniform as there is little information to guide clinicians on the optimal method of infection prophylaxis during B cell depletion and HGG. Though antibiotic prophylaxis and IgG replacement therapy have been reported to be effective, only 28% of centers used it routinely, 48% administering them in individual subjects [13, 18, 36]. This attitude seems to be rational, as serious infections requiring hospitalization were reported in less than 2.5% of the total number of RTX-treated children. In a long-term safety study of RTX in 2578 adults with rheumatoid arthritis, serious infections were reported in 7% [34]. Repeated courses of RTX, malignancies, concomitant steroid/IMS treatment, and pretreatment low IgG levels have been implicated as risk factors for significant HGG and severe infections [14, 15, 17, 18, 22, 29]. In a recent single-center Japanese study, the severity of HGG was not significantly associated with the risk of infection in nephrotic children [23].

Persistent HGG was recognized by a similar proportion of centers (47%) and in a similar overall number of children (128) as for pretreatment HGG. This suggests that, at least in some of the children, HGG may have already been present before RTX infusion and confirms the rationale for routine pretreatment evaluation of immunoglobulin levels. The prophylactic use of IGRT varied between units and only 1/3 units gave IGRT routinely for persistent HGG. Due to the survey design, it was not possible to assess the efficacy of IGRT. According to published recommendations for the management of HGG in children treated with B cell targeting therapy for rheumatic diseases, not all patients with HGG require supplementation [37]. Initiation of IGRT may be necessary in the presence of serious, persistent, unusual, or recurrent infections.

The number of 33 reported severe infections associated with the use of RTX in a total cohort of 1328 children with INS was comparatively low, confirming the relative safety of this biologic therapy for children with SDNS/FRNS.

Furthermore, the causal relationship of severe infections and RTX use is probably complex and additional factors may play a role including, but not limited to, previous or concomitant IMS drugs, pre-existing low IgG levels, or RTX dosage. The high frequency of HGG in subjects with severe infections (80%) is highly suggestive that low serum IgG contributes to the development of severe infections in a subset of nephrotic children, though most children with HGG seem to fare well [19, 23].

Though infrequent, serious infections in nephrotic subjects are an important issue and children with this complication require monitoring and rapid management as 3 deaths were recorded among the 33 children reported with severe infections.

In conclusion, we advocate for the routine screening for HGG prior to and post RTX treatment in children with FRNS/SDNS. The survey has demonstrated that if screened for, HGG is a widely recognized complication observed by nearly half of European pediatric nephrology centers using this off-label therapy. The main limitation of the performed survey is the lack of data on the exact incidence of HGG in children treated for INS and the influence of RTX and other IMS drugs on its incidence and duration. Furthermore, the design of the study did not enable a risk analysis for the development of HGG nor the risk of acquiring severe infections in its presence. Further studies are required before recommendations can be issued on the clinical significance and optimal management of HGG in children with idiopathic nephrotic syndrome who have received or will receive RTX.

## Supplementary Information

Graphical Abstract (PPTX 151 kb)

## Data Availability

The datasets generated and analyzed during the current study are available from the corresponding author on reasonable request.
