# Publisher Correction: Rituximab-associated hypogammaglobulinemia in children with idiopathic nephrotic syndrome: results of an ESPN survey

**DOI:** 10.1007/s00467-024-06625-w

**Published:** 2024-12-19

**Authors:** Aleksandra Zurowska, Magdalena Drozynska-Duklas, Rezan Topaloglu, Antonia Bouts, Olivia Boyer, Mohan Shenoy, Marina Vivarelli, ESPN Glomerulonephritis Working Group

**Affiliations:** 1 Department of Pediatrics, Nephrology and Hypertension, Medical University of Gdańsk, ul. Debinki 7, 80-952 Gdańsk, Poland; 2 Centre for Rare Diseases, Medical University of Gdańsk, Gdańsk, Poland; 3 Department of Pediatric Nephrology, Hacettepe University School of Medicine Hacettepe University, Ankara, Turkey; 4 Department of Pediatric Nephrology, Emma Children’s Hospital, Amsterdam University Medical Centers, Amsterdam, The Netherlands; 5 Department of Pediatric Nephrology, Reference Center for Idiopathic Nephrotic Syndrome in Children and Adults, Necker Hospital, Paris, France; 6 Laboratory of Hereditary Kidney Diseases, Imagine Institute, Paris Descartes University, Paris, France; 7 Department of Paediatric Nephrology, Royal Manchester Children’s Hospital, Manchester University Hospitals NHS Foundation Trust, Manchester Academic Health Science Centre, Manchester, UK; 8 Division of Nephrology and Dialysis, Bambino Gesù Children’s Hospital and Research Institute, Rome, Italy

**Publisher Correction: Pediatric Nephrology (2023) 38:3035–3042**

10.1007/s00467-023-05913-1

The original online HTML version of this article has been revised: The individual authors listed only in the acknowledgments were incorrectly added to the author line as collaborators representing the ESPN Glomerulonephritis Working Group. The following authors were incorrectly included:

K. Endén

A. Tsygin

R. Grenda

A. Raes

J.M. Van Hoeck Koen

B. Adams

M. Aksenova

B. Ranchin

N. Hooman

I. Ogarek

T. Seeman

M. Fila

L. Oni

S. Mir

R. Novo

S. Stabouli

J. Vara-Martín

J.A.E. van Wijk

R. Ehren

Z. Bekassy

M. Herthelius

F. Becherucci

H. Shasha-Lavsky

F. Santos

M. Feldkötter

M. Pańczyk-Tomaszewska

J. Harambat

D. Grima

I. Gökçe

A. Teixeira

C. Licht

I.M. Schmidt

P. Brandström

N. Dinçel

The original article has been corrected, and the publisher apologizes for the inconvenience.

