# Supplementary material for: Rituximab-associated hypogammaglobulinemia in children with idiopathic nephrotic syndrome: results of an ESPN survey
Source: Pediatr Nephrol. 2023 Apr 4;38(9):3035–42. doi: 10.1007/s00467-023-05913-1 (PMC10432325; doi:10.1007/s00467-023-05913-1)
Supplement: Supplementary file 1 — (PPTX 151 kb) [file 467_2023_5913_MOESM1_ESM.pptx]

## Slide 1
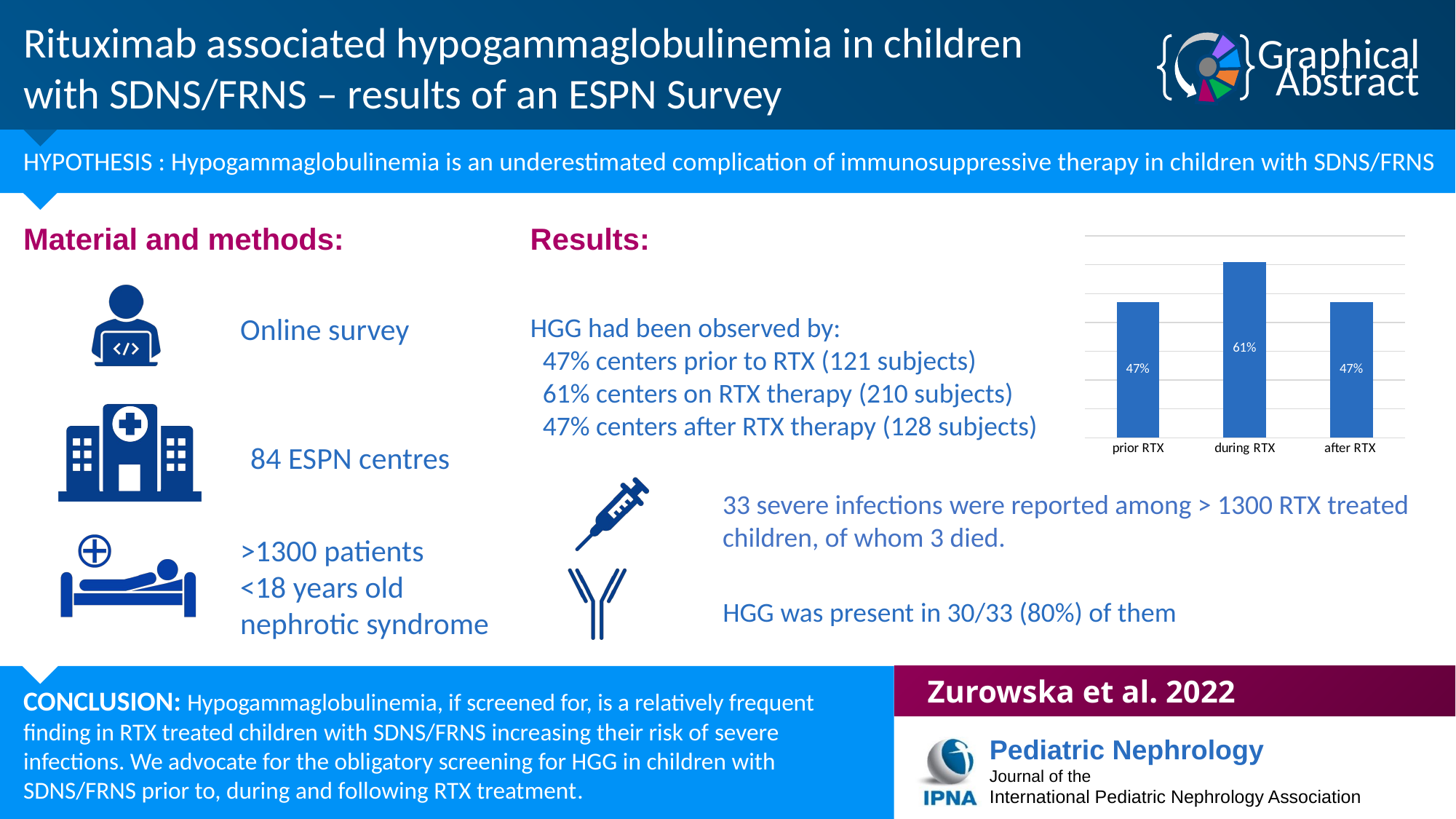

Rituximab associated hypogammaglobulinemia in children
with SDNS/FRNS – results of an ESPN Survey
HYPOTHESIS : Hypogammaglobulinemia is an underestimated complication of immunosuppressive therapy in children with SDNS/FRNS
Material and methods:
Results:
### Chart
| Category | |
|---|---|
| prior RTX | 0.47 |
| during RTX | 0.61 |
| after RTX | 0.47 |
Online survey
HGG had been observed by:
 47% centers prior to RTX (121 subjects)
 61% centers on RTX therapy (210 subjects)
 47% centers after RTX therapy (128 subjects)
84 ESPN centres
33 severe infections were reported among > 1300 RTX treated children, of whom 3 died.
>1300 patients
<18 years old
nephrotic syndrome
HGG was present in 30/33 (80%) of them
Zurowska et al. 2022
CONCLUSION: Hypogammaglobulinemia, if screened for, is a relatively frequent finding in RTX treated children with SDNS/FRNS increasing their risk of severe infections. We advocate for the obligatory screening for HGG in children with SDNS/FRNS prior to, during and following RTX treatment.
